# Bibliographical Notices

**Published:** 1854-09

**Authors:** 


### BIBLIOGRAPHICAL NOTICES.

*The Elements of Materia Medica and Therapeutics.* By JONATHAN PEREIRA, M.D., F.R.S., LL.S. *Third American Edition, Enlarged and Improved by the Author. Including Notices of most of the Medicinal Substances in use in the Civilized World, and forming an Encyclopædia of Materia Medica.* Edited by JOSEPH CARSON, M.D., Professor of Materia Medica in the University of Pennsylvania, etc. Vol. II.; Philadelphia, Blanchard & Lea, 1854; pp. 1226.

The large number of works for review which have been accumulating upon our hands for the last few months, have delayed the appearance of a notice of the voluminous second part of Pereira's Materia Medica, issued some months since by Messrs. Blanchard & Lea. As most of our readers are probably aware, this volume has been looked for and received with especial interest, owing to the lamented death of the author before its completion. The unfinished portion of this edition was entrusted to the hands of Drs. ALFRED SWAINE TAYLOR and GEORGE OWEN REES, of London, who profess, in the execution of their task, to "have in no case interfered with the views or opinions of the author," having "endeavored to act in accordance with his views, as embodied in a large collection of notes and memoranda which were entrusted to them for this purpose." They appear to have retained a large portion of the previous edition in an unaltered state. Some new matter of a useful character has been introduced. But in many particulars, the editorial revision has been of a hasty and inaccurate character, and has been the subject of general criticism.

We have already noticed the present edition of the first volume of this work, published some years since. The second volume is devoted exclusively to the consideration of Organic Substances, the Mineral Substances having been confined to the first volume. The additions in this edition are chiefly found in the second volume, and in all amount to four hundred pages of new matter. Three-fourths of the entire work had received the revision of the author, and, however we must regret his loss in the concluding portion, we must admit that it has in the main been presented to the profession with judgment and ability.

Of Dr. Pereira's great work on Materia Medica, the opinion of the medical profession throughout the world has been pronounced, without a dissenting voice, that it is without approach—facile princeps—the most complete treatise on the subject extant. Deficient, indeed, in profound or comprehensive views on therapeutics, still, in every detail connected with the history, characters, and application of drugs, it stands unrivalled in extent and precision of information, and must long be received as the standard work of reference on this branch of medicine. To use the language of his editors, Dr. Pereira may be said to have exhausted the subject of the Materia Medica, as we are at present acquainted with its articles, and his book constitutes truly a complete encyclopædia of this department. As a monument of erudition and research it is not surpassed, perhaps not equalled, in the annals of medicine.

At the time of Dr. Pereira's death, he had completed the article *Cinchona*, which has been entirely remodelled from the previous editions, and occupies sixty-four pages of the American edition. It is illustrated by numerous wood-cuts, and is a most complete monograph on this interesting pharmacological subject. Thirteen species of the genus cinchona are recognized by Dr. Pereira as yielding bark for commercial purposes. The arrangement of the barks according to their *color*, which has so long been generally adopted, and which was admitted by the author in his last edition, has been here abandoned as objectionable.

"The same species of bark (e. g. the bark of *C. lancifolia*) which in the young state, has a brown epidermis, is found, at a more advanced stage of its growth, to be whitish externally, owing to the exfoliation of its periderm, and the exposure of its white, micaceous, suberous coat. Moreover, the yellow or red color of the liber, on which is founded the distinction of yellow and red barks, cannot be relied on for characterizing any particular sort of bark; since the same species of bark may, under some circumstances, be red, under others yellow. Of this we have a good example in lancifolia bark.

"In a commercial point of view, the value of a cinchona bark depends on the quantity of quinine which it is capable of yielding; and an arrangement of barks founded on the nature of the alkaloid which they respectively contained, would be the most useful, both for commercial and medicinal purposes. But though, in a general way, a bark is termed a *quinine bark*, a *quinidine bark*, a *cinchonine bark*, or an *aricine bark*, yet cinchona barks cannot be correctly thus classified, because most cinchona barks contain two or three of these alkaloids, and differ from each other essentially in the relative proportion of these bases which they are capable of yielding. Their chemical distinction, therefore, is one rather of degree than absolute difference.

"In the absence of any scientific arrangement, the barks will be noticed in *geographical* order, commencing with the more valuable barks of the southern cinchona district (Bolivia) and proceeding northerly, finishing with the less valuable barks of the most northern cinchona district (New Granada.)

| | |
|---|---|
| Bolivia . . . | 1. Calisaya (*yellow*) bark. |
| Peru . . . | 2. Carabaya " " |
| | 3. Cusco " " |
| | 4. Huanuco (*gray*) " |
| | 5. Huamalies (*rusty*) " |
| | 6. Jaen (*ash*) " |
| Ecuador . . | 7. Loxa (*crown* and *pale*) bark. |
| | 8. Guayaquil (*red* and *pale*) bark. |
| New Granada | 9. Pitaya (*condaminea*) " |
| | 10. Bogota or Caqueta (*lance*) " |
| | 11. Carthagena (*leaved*) " |
| | 12. Maracaibo " |

Before describing the various kinds of cinchona barks met with in commerce, the author makes some extended remarks on their general characters, as their *cryptogamia*, *structure*, *fracture*, *markings*, *quilling*, *color*, *taste* and *odor*. At the time of his death, he was engaged in investigating the appearance of the barks, with a view of determining the seat of the *active principles*. The following observations will be read with interest:

"*Seat of the Active Principles.*—I have repeatedly submitted sections of Cinchona barks to microscopic examination, with the view of determining the seat and appearance of the alkaloids in their native tissues. The liber of many barks presents, even to the naked eye, a speckled appearance, owing to the presence of minute white spots. When we examine these spots by a low magnifying power, they are seen to be cells filled with a white solid substance. If we use the compound microscope, with an object glass of two inches focal length, the inner surface of the liber presents an amygdaloid appearance, owing to the presence of ovoid cells filled with a white substance, and imbedded in the yellowish brown tissue of the bark. Sometimes these cells are rectangular. Longitudinal and transverse sections of the bark show that these white masses are confined to the liber, and chiefly to its inner portion. In one specimen I discovered a thin layer of the white matter between the liber and the mesophlœum. These white masses I have met with more abundantly in the Cinchonine barks. When the white substance is submitted to high magnifying power, it appears like a crumbling mass, without presenting any distinct crystalline form. It is readily soluble in diluted hydrochloric acid, and the solution is not precipitated by oxalate of ammonia. It dissolves also in diluted sulphuric acid, and the solution by evaporation crystallizes. In alcohol, ether, and solution of caustic potash, it is only slightly soluble. It is probable, I think, that this white matter consists chiefly of some compound of the alkaloids of the bark."

The article on cinchona was concluded by the editors. On the subject of the adulterations of *sulphate of quinia*, the very common one now practised of sulphate of quinidia appears to have escaped their attention. As regards the *dose* for the administration of this article, it would, perhaps, have been well if the American editor had appended a note to the directions laid down in the English edition. Practitioners in the miasmatic districts of our country would find themselves not a little at fault if their dealings with the sulphate of quinia were characterized by the spirit of the following recommendation: "Disulphate of quinia is given in doses of from gr. j. to grs. v. Occasionally, it is exhibited in much larger doses as a febrifuge; but it is very apt to disagree, causing disturbance of stomach, febrile disorders, and headache. I have known fourteen grains taken, and have heard of a scruple, or half a drachm, being exhibited at a dose."

The British journals have pointed out a number of inaccuracies and omissions with which the editors are chargeable in the completion of Dr. Pereira's work. The London Pharmaceutical Journal thus sums them up:

In illustration of the omissions we complain of, we may refer to the article on *Amygdalus communis*, where it is stated that three varieties of the Sweet Almond are known in commerce, whereas in a paper published by the author in 1846, six varieties are described and figured.

More important omissions occur in the notice of Balsam of Peru, respecting which new and valuable information was brought forward by Dr. Pereira, in 1850. This, however, has been very sparingly, and not very correctly, used by the editors. We are told that the plant yielding the balsam grows in Peru, New Granada, Columbia and Mexico; whereas the author, in the Pharmaceutical Journal, vol. x., p. 285, states that the balsam is "exclusively the produce of the Balsam Coast, which extends from the Acajutla to the Port Libertad, on the Pacific side of Central America."

We are further informed, on the authority of one M. Victor le Nouvel, that "Peruvian Balsam always contains a good deal of water, sometimes as much as sixty per cent.," a statement so improbable, that we are surprised te see it repeated without comment.

White Balsam of Peru is briefly stated, on the authority of Guibourt, to be Solid Balsam of Liquidamber, while not the least allusion is made to the White Balsam of Sonsonate, which is derived from the seeds of the tree affording the so-called Black Balsam of Peru.

Moreover, no mention is made of Balsamito, which is prepared from the same tree as the Peruvian and White Balsams, all of which are described in the paper already referred to.

The promptitude with which the volume before us was completed, deserves commendation, but we observe a little indication of hasty revision in several places. Thus, in the notice of extract of Colocynth, no remark is made on the great difference in the products obtained by the London and Edinburgh processes, the statement of properties and dose being made to apply equally to both. In the account of Oil of Bitter Almonds, the notes which have been added, tend rather to confuse than to enlighten the reader, and do not, as we conceive, correctly represent the effects of the oil when deprived of hydrocyanic acid.

The editorial remark, introduced in the notice of *Spiritus Carui*, at page 1691, that "the simple solution of the oil, as recommended by the London College, is by far the best mode of preparing this and the other spirits of the Pharmacopœia," seems to indicate a want of practical knowledge and judgment in Pharmaceutical operations. We think Dr. Pereira would have said that the difficulty of obtaining the essential oils in an unadulterated state, and their great tendency, even if genuine, to become oxidized by exposure to the air, render the simple solution of the oils, without distillation, *by far the worst mode* of preparing the spirits of the Pharmacopœia.

It would, perhaps, be unreasonable to expect from the editors that vigilant watching of the drug market, by means of which the late Dr. Pereira was enabled to get the earliest intimation of any alteration that might occur in the importation or commerce of drugs. It was this practical knowledge, constantly renewed by intercourse with merchants and dealers, which so eminently qualified him as a writer of the natural history of drugs. Without it he could not have kept pace as he did with the progress of knowledge, or have represented so faithfully the commercial changes which are constantly occurring, in reference to the numerous subjects comprised in his great work. We have been reminded of the cessation of the accustomed vigilance in these respects, on referring to the article on Senna. The statement that Alexandrian Senna is "mixed *always* with the leaves of *Cynanchum Argel*," although correct at the date of the former edition, is not so at the present time; for since attention has been directed to it in the drug market, this adulteration has become rather the exception than the rule. We have recently examined several bales of Alexandrian Senna, in some of which none of the *Cynanchum Argel* was found; and in the others it was difficult to collect enough for a specimen.

We find also, on referring to the account of sulphate of quinine, that no mention is made of quinidine among the substances used for adulterating the quinine of commerce; yet this is almost the only adulteration met with, at the present time, in that article.

A note at page 1720, and similar notes in other places, would lead to the inference that the editors had forgotten the title and purport of the work they were editing. They say, "Although Opoponax no longer finds a place in any British Pharmacopœia, we have thought it desirable to retain the description given by the author; as, judging of the future by the past, it will probably be restored to the Materia Medica in a future edition of the Pharmacopœia." It might be inferred from this that Dr. Pereira's work was intended to comprise only the Materia Medica of the British Pharmacopœias, instead of including, as the title page represents, "notices of most of the medicinal substances in use in the civilized world, and forming an *Encyclopœdia of Materia Medica.*"

We must also advert to what appears to be a deviation from the principle announced by the editors in their preface as that by which they were guided in the introduction of new matter. They say that "they have in no case interfered with the views of the author." Yet in an editorial note appended to the notice of gelatine, at page 2232, it is stated: "Much absurd discussion has arisen as to whether gelatine is to be regarded in the light of a *product* or *educt* of the tissues. It is an educt of the swimming bladder of the sturgeon, and is properly described by the author (*infra*) as a *constituent* forming from 86 to 93 per cent. of isinglass. If an educt of the air-bladder of the sturgeon, it must be equally an educt of the skin of young animals, as of the calf, *i. e.*, it exists in the skin as such, and is not produced from it by the action of boiling water, any more than starch is produced from grain by a similar process." Now it is well known that, in common with other high authorities upon this subject, the late Dr. Pereira maintained that gelatine is a product and not an educt of the tissues; and we presume the discussion referred to in the above quotation was one in which he was opposed to one of the editors on this point. Little could he then have imagined that within so short a time his own book would be made the medium for designating his strongly advocated views as untenable and absurd. We hope to find this objectionable note omitted in the next edition, being uncalled for and inappropriate. The article headed "*Para Isinglass,*" is also, we think, misplaced in this work. The title is obviously wrong, the substance referred to having no claim to be called isinglass, of which it possesses none of the properties. A brief allusion to it as a substance imported more as a curiosity than with any view to commerce, would have been sufficient.

Among the editorial shortcomings which have been charged upon the volume under notice, perhaps one of the most flagrant is in the article *Chloroform.* In their account of this agent, Drs. Taylor and Reese make no mention of Dr. Simpson's discovery of its anæsthetic powers—an injustice which is so conspicuous and grievous, that it may be fairly laid to the account of personal hostility to the distinguished Edinburgh professor, and seriously affects the character of the editors for impartiality.

The additions of the American editor "pertain to the prominent vegetable productions of this country, and to the directions of the United States Pharmacopœia, in connection with all the articles contained in the volume which are referred to by it."

---

*The Pathology and Treatment of Stricture of the Urethra, both in the Male and Female; being the treatise for which the Jacksonian prize, for the year* 1852, *was awarded by the College of Surgeons in England.* By HENRY THOMPSON, F. R. C. S. M. B. London. Surgeon to the St. Marylebone General, and to the Blenheim Dispensaries; Fellow of the Medical and Chirurgical, and of the Pathological Societies; formerly House Surgeon to University College Hospital. 8vo. pp. 424. John Churchill. London. 1854.

Stricture of the urethra is a disease almost daily encountered by Surgeons in full practice; it is accompanied by much suffering, and is sometimes followed by serious results to the patient; it is difficult to cure, even by dexterous and experienced practitioners: for these reasons, it was wise in the "College of Surgeons of England" to select it, as a subject for a prize essay. And we may say at once, that, in our humble judgment, the work before us is a full equivalent for the prize, and in every respect worthy of approbation.

In the first chapter, the anatomy and physiology of the male urethra are accurately and minutely described. The classification and pathology; the symptoms and pathological effects; the causes of organic and permanent stricture; the pathology of strictures which are only of transient duration, are treated in the 2d, 3d, 4th and 5th chapters. Six chapters are devoted to the diagnosis and treatment of stricture of the urethra and its consequences in the male; and one chapter embraces an anatomical description of the female uretha, and an account of the symptoms and treatment of stricture in it.

The appendix contains a brief, but admirable essay on "the examination of urine for clinical purposes, chiefly in connexion with difficult micturition;" brief descriptions of pathological specimens illustrative of the subject, which may be found in the Museums of the Royal College of Surgeons; of Guy's Hospital; of Bartholomew's Hospital; of St. George's Hospital; of St. Thomas' Hospital; of University College; of Middlesex Hospital; of King's College Hospital; of the London Hospital, and of the Edinburgh Royal College of Surgeons: Reports of many cases; and a table, in which an analysis of two hundred and twenty cases of stricture, (exhibiting in each the age of the patient, "the antecedents and supposed causes," the "access and progress of the disease," and "present condition,") is succinctly given.

The text is illustrated by four plates and fifteen wood-cuts.

The plan observed in composing this essay is described by the author, in his preface, as follows:

"Firstly:—The observations and opinions of those writers who have paid especial attention to the subject, are, on most points, collated and adduced. In each case the writer's words are quoted, and direct reference is made to the page and edition of his book.

"Secondly:—Original researches have been made, as far as it has been within the author's means to do so, and their results are compared with the foregoing. Thus, the chapter on the pathological anatomy of stricture is mainly a digest of the facts now exhibited in the principal museums belonging to the medical schools of London, Edinburgh and Paris, in which *each preparation has been individually examined by himself* [the italics are ours.] A reference is made in the text to various specimens of importance, and an account of these is placed in the appendix, the bulk of which is thus somewhat increased, rather, however, for the purpose of facilitating the student's acquaintance with unquestionable examples and illustrations of the facts related than to furnish a body of matter possessing general interest and value.

"Thirdly:—In relation to the natural History, and to the treatment of stricture, a certain number of cases, hitherto unpublished, have been placed in the appendix, under the head of 'Reported cases' for the purpose of illustrating numerous points, connected with these divisions of the subject. Following these is a 'table of cases,' 220 in number, each containing a very brief statement of the chief incidents in the history of the patient, and his present condition, condensed from fully reported cases only, upon the aggregate of which have been founded, in a great measure, the chapters on 'The Symptoms' and on the 'Causes of Stricture.'

"It has been deemed necessary to discuss somewhat at length the 'quæstio vexata' of the present day, viz., that of cutting operations for stricture performed from the perineum. Certain data required for this purpose will be found under the head of 'Outlines of Cases,' which are merely very short histories, containing the principal facts bearing upon this question.

"Lastly:—Respecting, the anatomical relations of the normal as well as of the diseased urethra, no pains have been spared in order to develope the best practical mode of conveying, as far as this can be done on paper, sound information upon this important subject. It will be seen that a great number of bodies have been examined to supply the facts related. One, out of several illustrative preparations which were sent into the College of Surgeons with the Essay, contained portions of the corpus spongiosum from not less than twelve bodies to illustrate a point in its anatomy referred to at pages 38—41."

The inference, from a perusal of the work, is that the author is an able, well-trained observer, and a lover of truth. He has mastered the bibliography of the subject, and while he is careful to give due credit to others, he arrogates nothing.

As a sample of his style, and also to bring forward what struck us as an objectionable practice, we quote the following on the use of chloroform.

"The influence of chloroform I have sometimes found extremely useful in facilitating the passage of a catheter or sound through the urethra, especially when it is sensitive, and the pain produced by instrumental interference produces uncontrollable and involuntary efforts of resistance on the part of the patient. He should be rendered insensible in the usual manner and on some fresh occasion, not after previous unsuccessful attempts on the same day, and then no sound should be introduced until after he is fully under its influence. Let it be remembered that it is not for the purpose of permitting the instrument to be used with greater force than before, but in order to produce relaxation of the muscular tissues, both of the voluntary and involuntary kinds, that the chloroform is administered, and it must of course, be given to a sufficient extent to ensure this result."

The inhalation of chloroform is so extremely hazardous, and death has so frequently followed its use, that we should pause before we put the life of our patient in peril, with no other object than to render him insensible to a degree of pain which is tolerable.

The following is a summary of the author's views, in general, on the modes of treating stricture of the urethra.

"DILATATION has been found successful for the great majority of cases, but certainly inefficient for the cure of some old and extensive strictures, as well as for some which are accompanied by a highly irritable condition of the urethra.

"CAUTERIZATION must be regarded as a useful adjunct to dilatation in some few cases, especially in some of those in which a considerable degree of irritability exists. It is wholly inapplicable to the removal of old and extensive contractions.

"INTERNAL DIVISION is particularly suited to these last named cases, when situated in the anterior part of the urethra.

"There remain, therefore, by the process of exclusion, some *very irritable strictures* and *some obstinate and extensive ones;* the latter being usually situated about the junction of the spongy and the membranous portions, or a little anterior thereto, for which at present no adequate remedy has been described."—p. 234–5.

This brief notice is enough to indicate the nature and extent of the work, which will probably be regarded among the very best books for reference, on all points connected with stricture of the urethra. We cheerfully and cordially commend the volume, as a full compilation of what is known on the subject, to every member of the profession who desires to increase his knowledge of a complaint which often baffles the most skilful and experienced.

---

*The Dispensatory of the United States of America.* BY GEORGE B. WOOD, M. D., Professor of the Theory and Practice of Medicine in the University of Pennsylvania, &c.; and FRANKLIN BACHE, M. D., Professor of Chemistry in Jefferson Medical College of Philadelphia, &c. Tenth edition, carefully revised. Philadelphia: Lippincott, Grambo & Co. 1854.

The publication of the first edition of the above work constitutes an important epoch in the history of our medical literature. Previous to that period, "The Edinburgh New Dispensatory," and "The London Dispensatory" were the standard authorities in use. Reliable and excellent as these works were, they were, from their nativity, adapted mainly to the sphere of Great Britain, and were consequently deficient in much information that belonged almost exclusively to our own country. These reasons, and the fact that we had a national Pharmacopœia, which required an intelligent commentary, rendered an American Dispensatory a wide-felt desideratum, which was only temporarily and imperfectly supplied in Thacher's and Coxe's Dispensatories.

The profession in the United States is under a lasting debt of gratitude to Drs. Wood and Bache, who so ably remedied this deficiency. Conceived in an admirable spirit, distinguished by the excellent style of its composition, the accuracy of its facts, and its lucid explanation of chemical principles and processes; not too full or minute on any one subject, yet sufficiently elaborate for all practical purposes, and with extensive references to its various sources of knowledge, the United States Dispensatory embodied an amount of information on the topics of which it treated that could not easily be obtained from any other quarter. These qualities have justly rendered it so great a favorite with the profession, that, though both large and expensive, it has already passed through ten editions.

It would be considered a needless infliction upon the patience of our readers, were we to attempt any examination of the contents of the present work. That its authors have bestowed much labor upon it, to place it on a standard with the therapeutical and pharmaceutical discoveries made since the publication of the last edition is evident, even from an hasty inspection.

Among the additions made, we observe accounts of Chromic acid, Cotyledon umbilicus, Cucumber ointment, Cucurbita pepo, or pumpkin seeds, Fraxinus excelsior, Fruit essences, Gelseminum sempervirens, Guaco, Guano, Hydriodyc ether, Hydrocotyle asiatica, Iodoform, Lonicera caprifolium, Meat biscuit, Nitrate of copper, Nitroprusside of sodium, Phosphate of potassa, Sassy bark, Sulphate of nickel, Sulphite of soda, Tannate of alumina, Tellurium, Ultramarine, Urate of ammonia, Urea, Xanthorrhœa resins, &c. To secure a place for the description of these and other additions, the authors have been obliged to discard what seemed useless to them in the previous edition. They have, also, "found it necessary to increase somewhat the number of pages, as well as slightly to enlarge the dimensions of the page, by which means considerable space has been gained."

The publishers, Messrs. Lippincott, Grambo & Co., are entitled to praise for the execution of their part of the undertaking.

*Clinical Report on Dysentery, based on an Analysis of forty-nine cases, with Remarks on the Causation, Pathology, and Management of the Disease.* By Austin Flint, M. D.. Pp. 90. 1853.

*Clinical Report on Chronic Pleurisy, based on an Analysis of forty-seven cases.* By the same. Pp. 58.

It must be freely acknowledged, that medicine, as an art, fails to make commensurate progress with the accessory sciences. The reason appears to be owing to the intricate nature of the subject, and to the want of steadily pursuing the direct road of improving it.

Accurate observation and verified experience are doubtless the true foundation of the art; and had this foundation, laid by the fathers of medicine, been regularly built upon by succeeding physicians, it could not have remained as imperfect as it confessedly is. Few physicians have written a genuine, clear and historical account of their own practice.

The science of medicine, therefore, is greatly indebted to those who, during the period of its most rapid advancement are not seduced by the novelties that daily crowd upon their notice into the paths of brilliant speculation, but who devote their time and talents to bring forth their own experience on particular points of practice.

Dr. Flint in doing this, in the two excellent reports now before us, has put the profession under great obligations to him. These reports were originally published in the *Buffalo Medical Journal*, and are now reprinted with the hope "that they may prove acceptable to some who are not readers of the periodical just mentioned." After giving them a most careful perusal, we have formed a very high opinion of their value, not so much for the original matter they contain, as for the manner in which they are drawn up. To our mind they are models of medical writing, distinguished for simplicity, clearness and brevity; and bearing the impress of a practical mind which has long been habituated to accurate observation, and the formation of just and independent conclusions, unbiassed by prejudice, and unencumbered by hypothetical reasoning. The soundness of practice inculcated, and the simple and terse style in which it is set forth are such as must win the attention and instruct the mind, whilst they tend to infuse self-confidence in the learner.

We hesitate not to say that brief as these essays are, they are worth volumes of hasty and ill-digested generalization, making good the pithy saying of one of the writers of the memoirs of the French Academy of Surgery, that "the increase of knowledge is not like that of other things; for as our opportunities increase there is often a great diminution of bulk:" a precept on which medical writers would do well to ponder.

It is not our intention to make anything like a critical analysis of these reports, for we have not space to do so, and besides, this has already been done in another place by a far abler pen than ours; but as journalists we could not allow them to pass without drawing the attention of our readers to them, believing, as we do, that it is chiefly to this class of medical writing that medicine is to receive that aid which will enable it to take rank as a positive science.

---

*Institutions for the Insane, in Prussia, Austria and Germany.* By PLINY EARLE, M. D., &c., &c.

*An Examination of the practice of bloodletting in Mental Disorders.* By PLINY EARLE, M. D.

We have read, with unfeigned pleasure, the above works of Doctor Earle, containing as they do, most valuable information on the subject of insanity; a disease, though in itself of the utmost importance, the nature and treatment of which, is but very imperfectly understood by the great body of the profession.

In the volume on German asylums, Dr. Earle gives us a succinct, but lucid exposition of the periodical literature of "Psychiatrie;" the doctrines of the somatic, psycho-somatic and Psychic schools; comparative advantages of large and small establishments for the treatment of mental diseases; separate asylums for the curable and incurable insane; psychical instruction of medical students; clinics in hospitals for the insane: moral treatment of patients, &c. &c. The foregoing subjects are discussed in an introductory chapter; then follow separate descriptions of the architectural and sanitary arrangements, the physical and moral treatment pursued, number of patients, &c.

&c. of forty-nine public and eight private asylums, seventeen of which, were visited by the author.

The propriety of "Bloodletting in Mental Disorders" is discussed in a series of extracts from the writings of one hundred and twenty-five medical men, who have devoted special attention to the subject of insanity. A few of these advocate the use of the lancet, but the vast majority of living authors not only decry bloodletting as a remedy for insanity of itself, but assure us that when physical disease renders the abstraction of blood imperative, the quantity should be less, *cæteris paribus*, from a deranged than from a patient of sound mind. This is sound doctrine, and we cannot but thank Dr. Earle for placing it so distinctly and forcibly before the profession.

